# Di-μ-oxido-bis­({2,2′-[ethane-1,2-diylbis(nitrilo­methanylyl­idene)]diphen­olato}titanium(IV)) chloro­form disolvate

**DOI:** 10.1107/S1600536813029656

**Published:** 2013-11-06

**Authors:** Kirill V. Zaitsev, Sergey S. Karlov, Yulia A. Piskun, Irina V. Vasilenko, Andrei V. Churakov

**Affiliations:** a Department of Chemistry, M.V. Lomonosov Moscow State University, Leninskie Gory 1/3, Moscow 119991, Russian Federation; b Belarussian State University, Phys. Chem. Problems Res. Inst., 14 Leningradskaya St, Minsk 220030, Republic of Belarus; c Institute of General and Inorganic Chemistry, Russian Academy of Sciences, Leninskii Prosp. 31, Moscow 119991, Russian Federation

## Abstract

In the title structure, [Ti_2_(C_16_H_16_N_2_O_2_)_2_O_2_]·2CHCl_3_, the Ti atom is coordinated in a distorted octa­hedral geometry by the *O*,*N*,*N*′,*O*′ donor set of the salalen ligand and by two μ_2_-oxide O atoms, which bridge two Ti(salalen) fragments into a centrosymmetric dimeric unit. In the central Ti_2_(μ_2_-O)_2_ fragment, the metal–oxygen distances are significantly different [1.7962 (19) and 1.9292 (19) Å]. In the crystal, the chloro­form mol­ecule is anchored *via* an N—H⋯Cl and a bifurcated C—H⋯(O,O) hydrogen bond. Slipped π–π stacking [shortest C⋯C distance = 3.585 (4) Å] and C—H⋯π inter­actions contribute to the coherence of the structure.

## Related literature

For general background to the chemistry affording the tetra­dentate salalen ligand, see: Matsumoto *et al.* (2005 ▶, 2007 ▶). For the crystal structure of a salalen complex, see: Taylor *et al.* (2006 ▶). For the structure of the parent titanium salen compound, see: Tsuchimoto (2001 ▶). For our previous work on titanium(IV) complexes with polydentate *N*,*O*-ligands, see: Zaitsev *et al.* (2006 ▶, 2008 ▶).
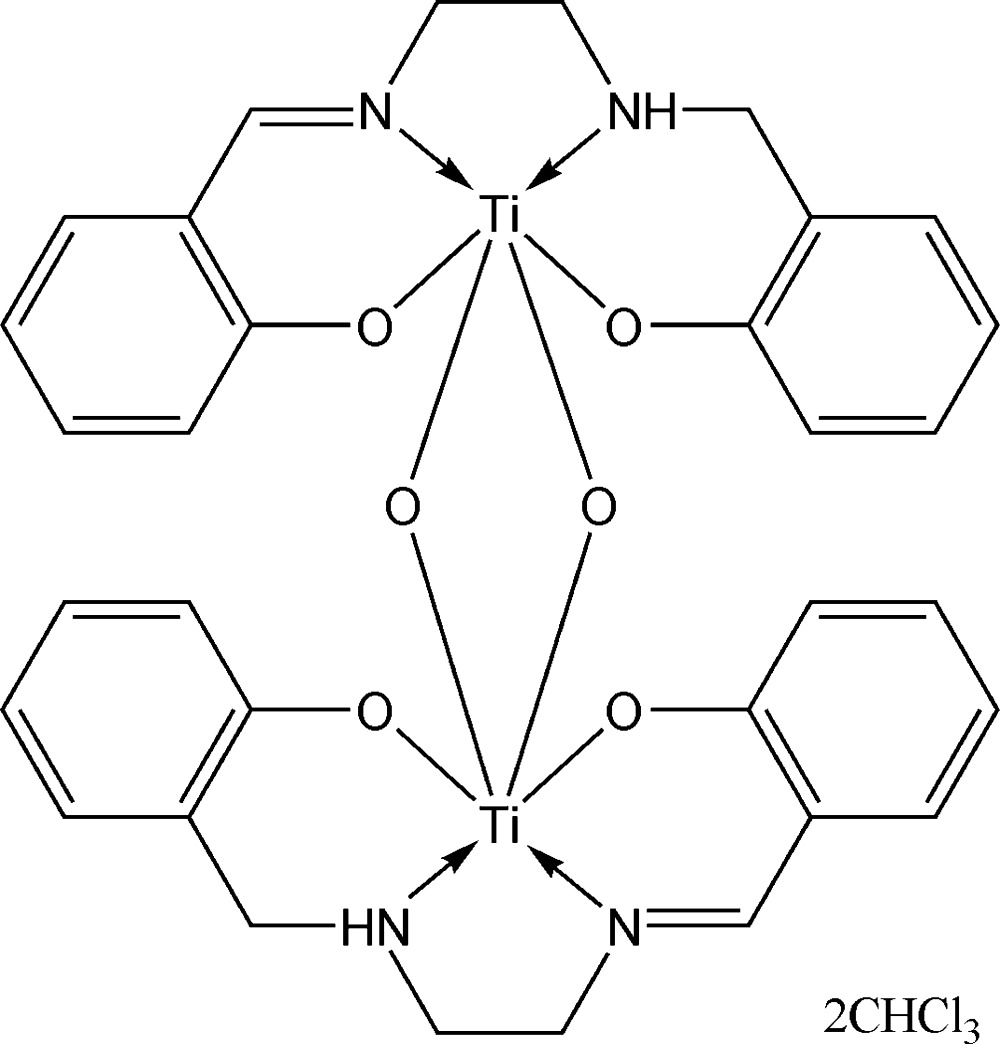


## Experimental

### 

#### Crystal data

[Ti_2_(C_16_H_16_N_2_O_2_)_2_O_2_]·2CHCl_3_
*M* *_r_* = 903.15
Triclinic, 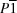

*a* = 10.237 (3) Å
*b* = 10.356 (3) Å
*c* = 10.936 (3) Å
α = 117.075 (4)°
β = 93.113 (4)°
γ = 110.463 (4)°
*V* = 935.0 (4) Å^3^
*Z* = 1
Mo *K*α radiation
μ = 0.91 mm^−1^
*T* = 150 K
0.08 × 0.06 × 0.01 mm

#### Data collection

Bruker SMART APEXII diffractometer
Absorption correction: multi-scan (*SADABS*; Bruker, 2008 ▶) *T*_min_ = 0.931, *T*_max_ = 0.991
8213 measured reflections
3668 independent reflections
2761 reflections with *I* > 2σ(*I*)
*R*_int_ = 0.037

#### Refinement

*R*[*F* ^2^ > 2σ(*F* ^2^)] = 0.041
*wR*(*F* ^2^) = 0.092
*S* = 1.03
3668 reflections
238 parameters
H atoms treated by a mixture of independent and constrained refinement
Δρ_max_ = 0.41 e Å^−3^
Δρ_min_ = −0.43 e Å^−3^

### 

Data collection: *APEX2* (Bruker, 2008 ▶); cell refinement: *SAINT* (Bruker, 2008 ▶); data reduction: *SAINT*; program(s) used to solve structure: *SHELXTL* (Sheldrick, 2008 ▶); program(s) used to refine structure: *SHELXTL*; molecular graphics: *SHELXTL*; software used to prepare material for publication: *SHELXTL*.

## Supplementary Material

Crystal structure: contains datablock(s) I. DOI: 10.1107/S1600536813029656/qk2062sup1.cif

Structure factors: contains datablock(s) I. DOI: 10.1107/S1600536813029656/qk2062Isup2.hkl

Supplementary material file. DOI: 10.1107/S1600536813029656/qk2062Isup3.mol

969061

Additional supplementary materials:  crystallographic information; 3D view; checkCIF report

## Figures and Tables

**Table 1:** Hydrogen-bond geometry (Å, °) *Cg*1 and *Cg*2 are the centroids of the C11–C16 and C21–C26 rings, respectively.

*D*—H⋯*A*	*D*—H	H⋯*A*	*D*⋯*A*	*D*—H⋯*A*
N2—H2⋯Cl3^i^	0.81 (3)	2.84 (3)	3.575 (3)	151 (3)
C1—H1⋯O2	1.00	2.55	3.506 (4)	160
C1—H1⋯O3	1.00	2.51	3.257 (4)	131
C17—H17⋯*Cg*2^ii^	0.95	2.81	3.754 (4)	174
C23—H23⋯*Cg*1^iii^	0.95	2.86	3.747 (4)	156

Symmetry codes: (i) 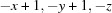; (ii) 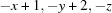; (iii) 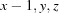.

**Fig. 1.**
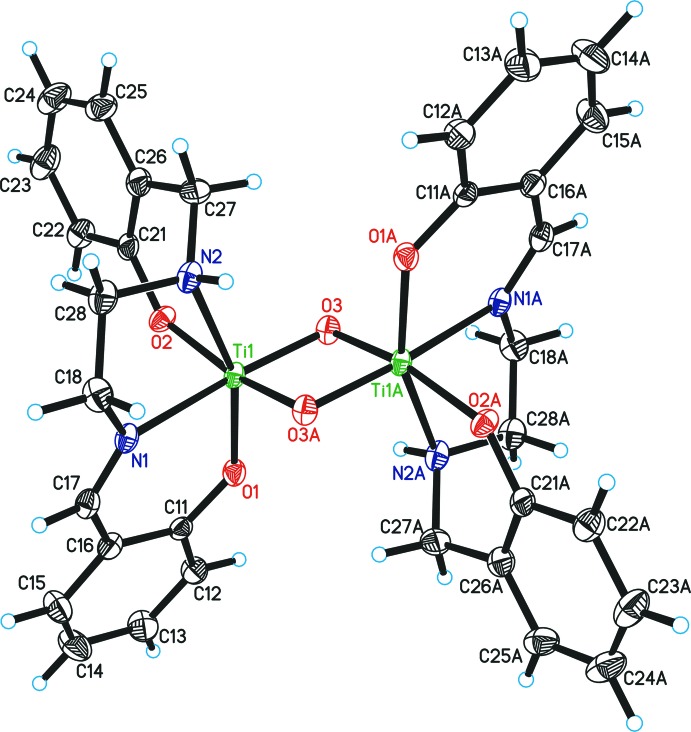
The molecular structure of the title compound, showing the numbering scheme adopted. Displacement ellipsoids are shown at the 50% probability level. Hydrogen atoms are omitted for clarity. Trailing A in the atom labels indicates symmetry transformation 1-x, 2-y, 1-z.

## supplementary crystallographic information

### 1. Comment

As a part of our investigation on chemistry of titanium complexes based on tridentate or tetradentate ligands (Zaitsev *et al.*, 2006, 2008) we obtained and studied the structure of the titanium compound [(salalen)TiO]_2_.

The title titanium salalen complex is centrosymmetric. Both Ti atoms are linked by µ_2_-oxo briges and possess a distorted octahedral coordination environment with *cis* interligand angles ranging from 81.27 (9) to 101.11 (8)°. In the central Ti_2_(µ^2^-O)_2_ fragment, metal-oxygen distances are significantly different (1.7962 (19) and 1.9292 (19) Å).

In the crystal, the solvent chloroform molecule forms bifurcated C—H···O hydrogen bond with the main molecule with C···O separations of 3.257 (4) and 3.506 (4) Å.

To the best of our knowledge, the title compound represents the second example of structurally characterized salalen complex (Taylor *et al.*, 2006).

### 2. Experimental

The several crystals of the title salalen complex were unexpectedly obtained after attempt to recrystallize the parent [(salalen)TiO]_2_ (Tsuchimoto, 2001) compound from a hexane-chloroform mixture.

### 3. Refinement

Amine hydrogen atom H2 was found from difference Fourier synthesis and its positional parameters were refined using *U*_iso_(H2) as 1.2*U*_eq_ of the parent nitrogen atom. All other hydrogen atoms were placed in calculated positions and refined using a riding model with C—H = 0.95 – 1.00 Å and *U*_iso_(H) = 1.2*U*_eq_(C).

### Crystal data

**Table:**

[Ti_2_(C_16_H_16_N_2_O_2_)_2_O_2_]·2CHCl_3_	*Z* = 1
*M**_r_* = 903.15	*F*(000) = 460
Triclinic, *P*1	*D*_x_ = 1.604 Mg m^−^^3^
Hall symbol: -P 1	Mo *K*α radiation, λ = 0.71073 Å
*a* = 10.237 (3) Å	Cell parameters from 1919 reflections
*b* = 10.356 (3) Å	θ = 2.2–25.7°
*c* = 10.936 (3) Å	µ = 0.91 mm^−^^1^
α = 117.075 (4)°	*T* = 150 K
β = 93.113 (4)°	Plate, light-yellow
γ = 110.463 (4)°	0.08 × 0.06 × 0.01 mm
*V* = 935.0 (4) Å^3^	

### Data collection

**Table:**

Bruker SMART APEXII diffractometer	3668 independent reflections
Radiation source: fine-focus sealed tube	2761 reflections with *I* > 2σ(*I*)
Graphite monochromator	*R*_int_ = 0.037
ω scans	θ_max_ = 26.0°, θ_min_ = 2.2°
Absorption correction: multi-scan (*SADABS*; Bruker, 2008)	*h* = −12→12
*T*_min_ = 0.931, *T*_max_ = 0.991	*k* = −12→12
8213 measured reflections	*l* = −13→13

### Refinement

**Table:**

Refinement on *F*^2^	Primary atom site location: structure-invariant direct methods
Least-squares matrix: full	Secondary atom site location: difference Fourier map
*R*[*F*^2^ > 2σ(*F*^2^)] = 0.041	Hydrogen site location: difference Fourier map
*wR*(*F*^2^) = 0.092	H atoms treated by a mixture of independent and constrained refinement
*S* = 1.03	*w* = 1/[σ^2^(*F*_o_^2^) + (0.0393*P*)^2^ + 0.3887*P*] where *P* = (*F*_o_^2^ + 2*F*_c_^2^)/3
3668 reflections	(Δ/σ)_max_ < 0.001
238 parameters	Δρ_max_ = 0.41 e Å^−^^3^
0 restraints	Δρ_min_ = −0.43 e Å^−^^3^

### Special details

**Table:**

Geometry. All e.s.d.'s (except the e.s.d. in the dihedral angle between two l.s. planes) are estimated using the full covariance matrix. The cell e.s.d.'s are taken into account individually in the estimation of e.s.d.'s in distances, angles and torsion angles; correlations between e.s.d.'s in cell parameters are only used when they are defined by crystal symmetry. An approximate (isotropic) treatment of cell e.s.d.'s is used for estimating e.s.d.'s involving l.s. planes.
Refinement. Refinement of *F*^2^ against ALL reflections. The weighted *R*-factor *wR* and goodness of fit *S* are based on *F*^2^, conventional *R*-factors *R* are based on *F*, with *F* set to zero for negative *F*^2^. The threshold expression of *F*^2^ > σ(*F*^2^) is used only for calculating *R*-factors(gt) *etc*. and is not relevant to the choice of reflections for refinement. *R*-factors based on *F*^2^ are statistically about twice as large as those based on *F*, and *R*-factors based on ALL data will be even larger.

### Fractional atomic coordinates and isotropic or equivalent isotropic displacement parameters (Å^2^)

**Table:**

	*x*	*y*	*z*	*U*_iso_*/*U*_eq_	
Ti1	0.50658 (5)	0.65403 (6)	0.05634 (5)	0.01468 (14)	
O1	0.6684 (2)	0.7828 (2)	0.21655 (19)	0.0185 (4)	
O2	0.3932 (2)	0.7673 (2)	0.13984 (19)	0.0186 (4)	
O3	0.4197 (2)	0.4842 (2)	0.07711 (19)	0.0179 (4)	
N1	0.6242 (2)	0.8318 (3)	−0.0058 (2)	0.0166 (5)	
N2	0.3580 (3)	0.5823 (3)	−0.1413 (3)	0.0198 (5)	
H2	0.374 (3)	0.509 (4)	−0.198 (3)	0.030*	
C11	0.7673 (3)	0.9337 (3)	0.2842 (3)	0.0166 (6)	
C12	0.8403 (3)	1.0016 (3)	0.4247 (3)	0.0217 (6)	
H12	0.8190	0.9395	0.4696	0.026*	
C13	0.9432 (3)	1.1579 (4)	0.4998 (3)	0.0285 (7)	
H13	0.9911	1.2026	0.5960	0.034*	
C14	0.9772 (3)	1.2501 (4)	0.4359 (3)	0.0328 (8)	
H14	1.0475	1.3578	0.4879	0.039*	
C15	0.9077 (3)	1.1837 (4)	0.2962 (3)	0.0274 (7)	
H15	0.9326	1.2463	0.2519	0.033*	
C16	0.8012 (3)	1.0262 (3)	0.2176 (3)	0.0191 (6)	
C17	0.7329 (3)	0.9660 (3)	0.0716 (3)	0.0186 (6)	
H17	0.7713	1.0303	0.0308	0.022*	
C18	0.5634 (3)	0.7824 (4)	−0.1528 (3)	0.0226 (7)	
H18A	0.5968	0.7047	−0.2195	0.027*	
H18B	0.5944	0.8762	−0.1659	0.027*	
C21	0.2627 (3)	0.7615 (3)	0.1055 (3)	0.0190 (6)	
C22	0.2212 (3)	0.8734 (4)	0.2051 (3)	0.0236 (7)	
H22	0.2850	0.9515	0.2964	0.028*	
C23	0.0879 (3)	0.8713 (4)	0.1718 (4)	0.0303 (8)	
H23	0.0617	0.9490	0.2401	0.036*	
C24	−0.0073 (3)	0.7581 (4)	0.0410 (4)	0.0323 (8)	
H24	−0.0991	0.7565	0.0192	0.039*	
C25	0.0326 (3)	0.6466 (4)	−0.0583 (3)	0.0268 (7)	
H25	−0.0333	0.5679	−0.1484	0.032*	
C26	0.1668 (3)	0.6469 (3)	−0.0294 (3)	0.0210 (6)	
C27	0.2045 (3)	0.5213 (3)	−0.1398 (3)	0.0243 (7)	
H27A	0.1818	0.4316	−0.1214	0.029*	
H27B	0.1437	0.4789	−0.2346	0.029*	
C28	0.4012 (3)	0.7066 (3)	−0.1807 (3)	0.0232 (7)	
H28A	0.3675	0.7892	−0.1244	0.028*	
H28B	0.3562	0.6586	−0.2825	0.028*	
C1	0.4183 (3)	0.6758 (4)	0.4095 (3)	0.0325 (8)	
H1	0.4220	0.6818	0.3210	0.039*	
Cl1	0.26515 (9)	0.50389 (11)	0.37249 (10)	0.0455 (3)	
Cl2	0.40908 (11)	0.84731 (11)	0.54051 (11)	0.0507 (3)	
Cl3	0.57585 (8)	0.66277 (9)	0.46628 (8)	0.0301 (2)	

### Atomic displacement parameters (Å^2^)

**Table:**

	*U*^11^	*U*^22^	*U*^33^	*U*^12^	*U*^13^	*U*^23^
Ti1	0.0197 (3)	0.0154 (3)	0.0121 (3)	0.0095 (2)	0.0039 (2)	0.0078 (2)
O1	0.0219 (11)	0.0170 (10)	0.0174 (10)	0.0081 (9)	0.0017 (8)	0.0096 (9)
O2	0.0198 (11)	0.0202 (10)	0.0149 (10)	0.0119 (9)	0.0027 (8)	0.0059 (9)
O3	0.0226 (11)	0.0189 (10)	0.0172 (10)	0.0111 (9)	0.0089 (8)	0.0110 (9)
N1	0.0229 (13)	0.0180 (12)	0.0131 (12)	0.0119 (11)	0.0051 (10)	0.0085 (11)
N2	0.0263 (14)	0.0197 (13)	0.0166 (13)	0.0136 (12)	0.0053 (11)	0.0086 (11)
C11	0.0152 (14)	0.0162 (14)	0.0186 (15)	0.0091 (12)	0.0042 (12)	0.0072 (12)
C12	0.0233 (16)	0.0232 (16)	0.0188 (15)	0.0084 (13)	0.0025 (13)	0.0121 (13)
C13	0.0305 (18)	0.0246 (17)	0.0209 (16)	0.0071 (15)	−0.0028 (14)	0.0086 (14)
C14	0.0296 (19)	0.0222 (17)	0.0306 (19)	−0.0002 (15)	−0.0005 (15)	0.0101 (15)
C15	0.0301 (18)	0.0219 (16)	0.0273 (17)	0.0049 (14)	0.0054 (14)	0.0150 (14)
C16	0.0204 (15)	0.0196 (15)	0.0182 (15)	0.0107 (13)	0.0029 (12)	0.0088 (13)
C17	0.0230 (16)	0.0213 (16)	0.0189 (15)	0.0133 (14)	0.0091 (13)	0.0126 (13)
C18	0.0290 (17)	0.0245 (16)	0.0170 (15)	0.0103 (14)	0.0070 (13)	0.0131 (13)
C21	0.0198 (16)	0.0210 (15)	0.0254 (16)	0.0096 (13)	0.0081 (13)	0.0179 (14)
C22	0.0255 (17)	0.0278 (17)	0.0234 (16)	0.0147 (14)	0.0094 (13)	0.0148 (14)
C23	0.0337 (19)	0.043 (2)	0.0357 (19)	0.0273 (17)	0.0198 (16)	0.0273 (17)
C24	0.0239 (18)	0.053 (2)	0.043 (2)	0.0217 (17)	0.0167 (16)	0.0374 (19)
C25	0.0199 (16)	0.0364 (18)	0.0319 (18)	0.0100 (14)	0.0061 (14)	0.0246 (16)
C26	0.0228 (16)	0.0233 (16)	0.0232 (16)	0.0100 (14)	0.0069 (13)	0.0164 (14)
C27	0.0201 (16)	0.0229 (16)	0.0228 (16)	0.0049 (13)	−0.0041 (13)	0.0102 (14)
C28	0.0301 (17)	0.0268 (16)	0.0180 (15)	0.0137 (14)	0.0055 (13)	0.0143 (14)
C1	0.0328 (19)	0.053 (2)	0.0273 (18)	0.0241 (17)	0.0141 (15)	0.0276 (17)
Cl1	0.0276 (5)	0.0486 (6)	0.0434 (5)	0.0112 (4)	0.0027 (4)	0.0147 (5)
Cl2	0.0553 (6)	0.0449 (6)	0.0706 (7)	0.0339 (5)	0.0283 (5)	0.0328 (5)
Cl3	0.0289 (4)	0.0344 (5)	0.0295 (4)	0.0154 (4)	0.0090 (3)	0.0164 (4)

### Geometric parameters (Å, º)

**Table:**

Ti1—O3	1.7962 (19)	C16—C17	1.447 (4)
Ti1—O1	1.8991 (19)	C17—H17	0.9500
Ti1—O2	1.9102 (19)	C18—C28	1.511 (4)
Ti1—O3^i^	1.9292 (19)	C18—H18A	0.9900
Ti1—N2	2.220 (2)	C18—H18B	0.9900
Ti1—N1	2.232 (2)	C21—C22	1.399 (4)
Ti1—Ti1^i^	2.7958 (12)	C21—C26	1.408 (4)
O1—C11	1.332 (3)	C22—C23	1.383 (4)
O2—C21	1.341 (3)	C22—H22	0.9500
O3—Ti1^i^	1.9292 (19)	C23—C24	1.376 (5)
N1—C17	1.277 (3)	C23—H23	0.9500
N1—C18	1.468 (3)	C24—C25	1.384 (4)
N2—C28	1.471 (4)	C24—H24	0.9500
N2—C27	1.479 (4)	C25—C26	1.392 (4)
N2—H2	0.81 (3)	C25—H25	0.9500
C11—C12	1.393 (4)	C26—C27	1.507 (4)
C11—C16	1.414 (4)	C27—H27A	0.9900
C12—C13	1.382 (4)	C27—H27B	0.9900
C12—H12	0.9500	C28—H28A	0.9900
C13—C14	1.386 (4)	C28—H28B	0.9900
C13—H13	0.9500	C1—Cl2	1.742 (3)
C14—C15	1.378 (4)	C1—Cl1	1.765 (3)
C14—H14	0.9500	C1—Cl3	1.767 (3)
C15—C16	1.403 (4)	C1—H1	1.0000
C15—H15	0.9500		
			
O3—Ti1—O1	101.11 (8)	C15—C16—C11	118.5 (3)
O3—Ti1—O2	98.43 (9)	C15—C16—C17	118.2 (3)
O1—Ti1—O2	95.69 (8)	C11—C16—C17	123.4 (3)
O3—Ti1—O3^i^	82.80 (9)	N1—C17—C16	124.0 (3)
O1—Ti1—O3^i^	100.23 (8)	N1—C17—H17	118.0
O2—Ti1—O3^i^	163.49 (8)	C16—C17—H17	118.0
O3—Ti1—N2	100.65 (9)	N1—C18—C28	107.2 (2)
O1—Ti1—N2	158.23 (9)	N1—C18—H18A	110.3
O2—Ti1—N2	81.27 (9)	C28—C18—H18A	110.3
O3^i^—Ti1—N2	82.33 (9)	N1—C18—H18B	110.3
O3—Ti1—N1	168.80 (8)	C28—C18—H18B	110.3
O1—Ti1—N1	82.93 (8)	H18A—C18—H18B	108.5
O2—Ti1—N1	91.50 (8)	O2—C21—C22	119.4 (3)
O3^i^—Ti1—N1	86.21 (8)	O2—C21—C26	121.7 (3)
N2—Ti1—N1	75.64 (9)	C22—C21—C26	118.9 (3)
O3—Ti1—Ti1^i^	43.20 (6)	C23—C22—C21	120.6 (3)
O1—Ti1—Ti1^i^	104.27 (6)	C23—C22—H22	119.7
O2—Ti1—Ti1^i^	139.09 (7)	C21—C22—H22	119.7
O3^i^—Ti1—Ti1^i^	39.60 (5)	C24—C23—C22	120.8 (3)
N2—Ti1—Ti1^i^	91.53 (7)	C24—C23—H23	119.6
N1—Ti1—Ti1^i^	125.78 (6)	C22—C23—H23	119.6
C11—O1—Ti1	136.03 (17)	C23—C24—C25	119.2 (3)
C21—O2—Ti1	138.85 (18)	C23—C24—H24	120.4
Ti1—O3—Ti1^i^	97.20 (9)	C25—C24—H24	120.4
C17—N1—C18	119.5 (2)	C24—C25—C26	121.6 (3)
C17—N1—Ti1	127.32 (19)	C24—C25—H25	119.2
C18—N1—Ti1	113.18 (17)	C26—C25—H25	119.2
C28—N2—C27	113.9 (2)	C25—C26—C21	118.9 (3)
C28—N2—Ti1	112.28 (17)	C25—C26—C27	119.7 (3)
C27—N2—Ti1	112.94 (17)	C21—C26—C27	121.3 (2)
C28—N2—H2	107 (2)	N2—C27—C26	113.1 (2)
C27—N2—H2	109 (2)	N2—C27—H27A	109.0
Ti1—N2—H2	100 (2)	C26—C27—H27A	109.0
O1—C11—C12	118.7 (2)	N2—C27—H27B	109.0
O1—C11—C16	122.2 (2)	C26—C27—H27B	109.0
C12—C11—C16	119.1 (3)	H27A—C27—H27B	107.8
C13—C12—C11	120.9 (3)	N2—C28—C18	109.8 (2)
C13—C12—H12	119.5	N2—C28—H28A	109.7
C11—C12—H12	119.5	C18—C28—H28A	109.7
C12—C13—C14	120.5 (3)	N2—C28—H28B	109.7
C12—C13—H13	119.7	C18—C28—H28B	109.7
C14—C13—H13	119.7	H28A—C28—H28B	108.2
C15—C14—C13	119.2 (3)	Cl2—C1—Cl1	110.39 (17)
C15—C14—H14	120.4	Cl2—C1—Cl3	109.84 (18)
C13—C14—H14	120.4	Cl1—C1—Cl3	109.57 (18)
C14—C15—C16	121.7 (3)	Cl2—C1—H1	109.0
C14—C15—H15	119.2	Cl1—C1—H1	109.0
C16—C15—H15	119.2	Cl3—C1—H1	109.0

Symmetry code: (i) −*x*+1, −*y*+1, −*z*.

### Hydrogen-bond geometry (Å, º)

Cg1 and Cg2 are the centroids of the C11–C16 and C21–C26 rings, respectively.

**Table:**

*D*—H···*A*	*D*—H	H···*A*	*D*···*A*	*D*—H···*A*
N2—H2···Cl3^i^	0.81 (3)	2.84 (3)	3.575 (3)	151 (3)
C1—H1···O2	1.00	2.55	3.506 (4)	160
C1—H1···O3	1.00	2.51	3.257 (4)	131
C17—H17···*Cg*2^ii^	0.95	2.81	3.754 (4)	174
C23—H23···*Cg*1^iii^	0.95	2.86	3.747 (4)	156

Symmetry codes: (i) −*x*+1, −*y*+1, −*z*; (ii) −*x*+1, −*y*+2, −*z*; (iii) *x*−1, *y*, *z*.
